# Supplementary material for: Molecular mechanism of Gαi activation by non-GPCR proteins with a Gα-Binding and Activating motif
Source: Nat Commun. 2017 May 18;8:15163. doi: 10.1038/ncomms15163 (PMC5454376; doi:10.1038/ncomms15163)
Supplement: Supplementary Information — Supplementary figures, supplementary notes and supplementary references. [file ncomms15163-s1.pdf]

SUPPLEMENTARY FIGURE 1

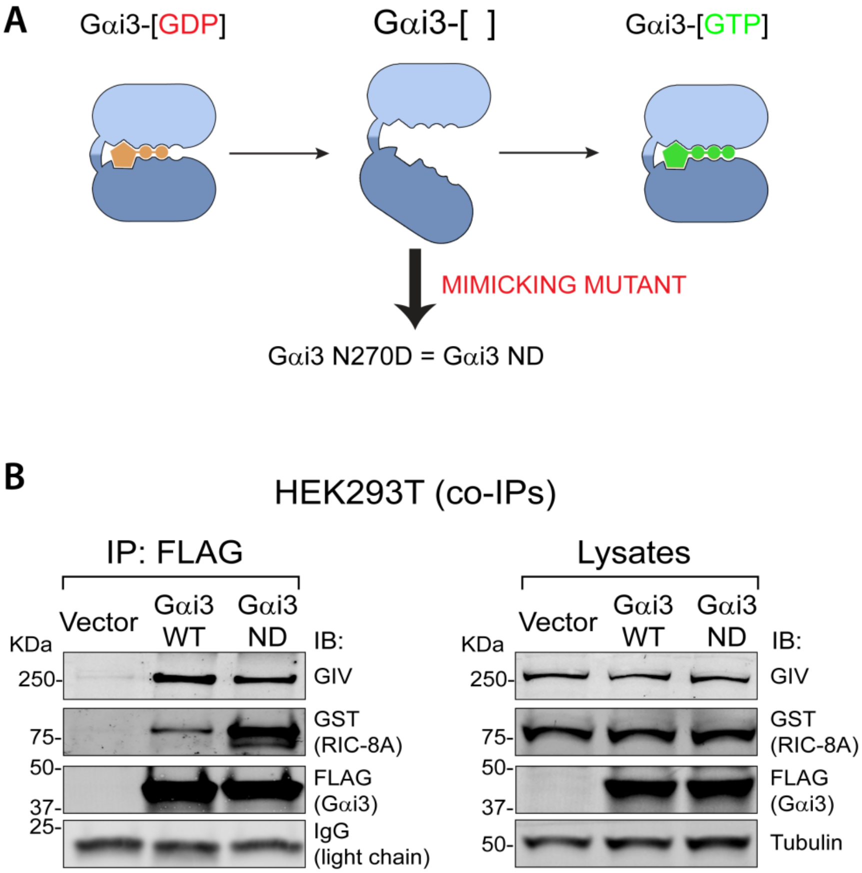

Supplementary Fig. 1. GIV binding to Gαi3 is not enhanced by a mutation that mimics the nucleotide-free G protein.

A. Gαi3-[ ]-mimicking mutant. Scheme depicting three different conformations of Gαi3 along the activation pathway (Gαi3-[GDP], Gαi3-[ ] and Gαi3-[GTP]) and a previously described mutant (Gαi3 N269D) that mimics the nucleotide-free conformation and works as a dominant-negative by binding irreversibly to GPCR GEFs[1].

B. GIV binds similarly to Gαi3 WT and the Gαi3-[ ]-mimicking mutant Gαi3 ND in co-immunoprecipitation assays. HEK293T cells were transfected with FLAG-tagged Gαi3 WT and Gαi3 ND and lysates immunoprecipitated with anti-FLAG antibodies. Immunoprecipitates (IP) are shown on the left and equal aliquots of the cell lysates are shown on the right. Gαi3 WT and Gαi3 ND were expressed and immunoprecipitated at similar levels. Ric-8A binds more Gαi3 ND than Gαi3 WT, validating that the mutant mimics Gαi3-[ ] under these experimental conditions. One experiment representative of at least 3 is shown for each panel. IgG= Immunoglobulin G.

SUPPLEMENTARY FIGURE 2

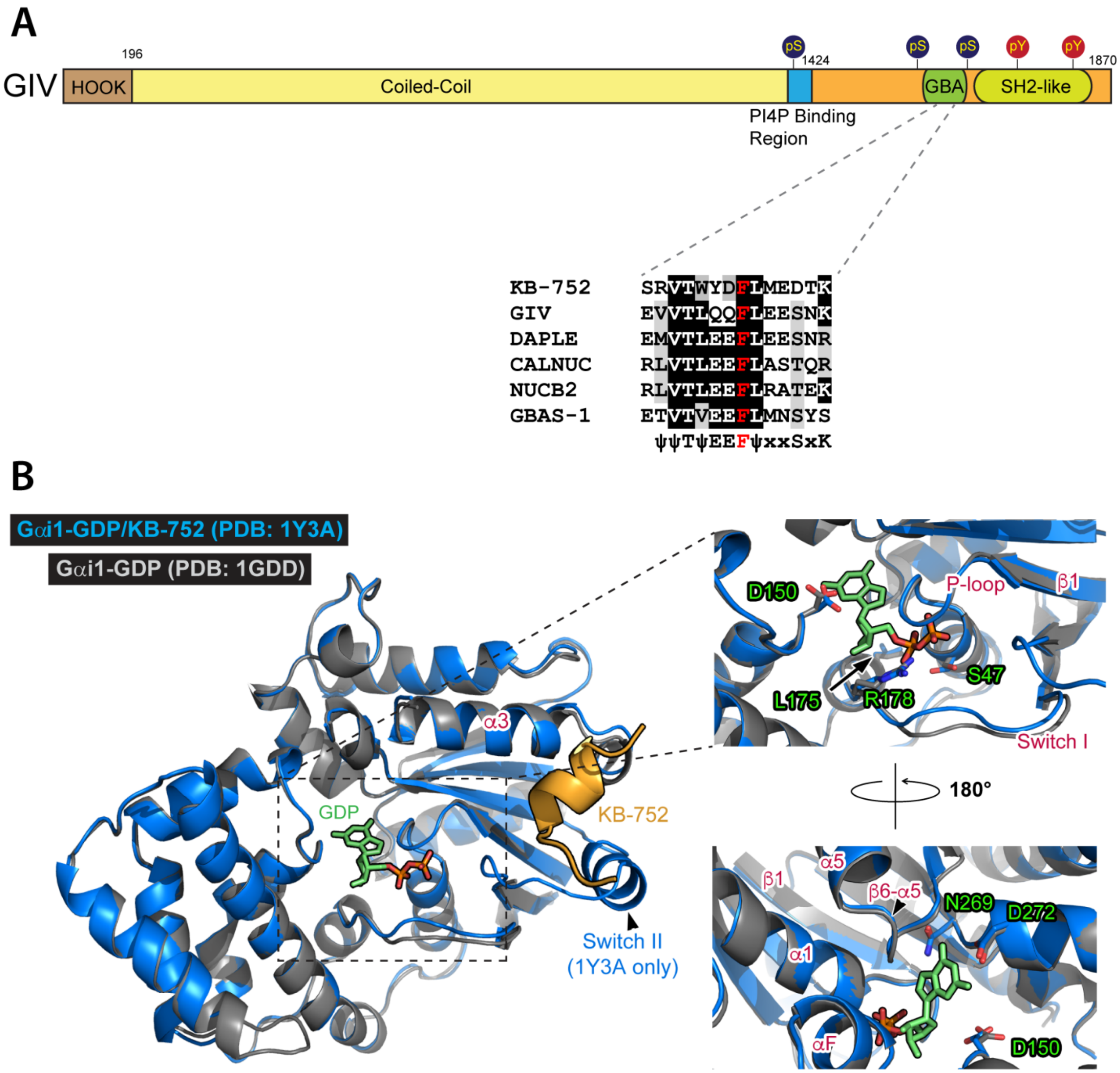

Supplementary Fig. 2. Sequence similarity between the GBA motif and KB-752, a non-native peptide crystallized in complex with Gαi1.

A. *Top,* scheme of GIV domains with phosphorylation sites. The HOOK domain has been proposed to mediate interaction with microtubules[2], whereas the coiled-coil domain might be responsible for dimerization[3]. The function of the phosphoinositide (PI4P) binding region is regulated by serine phosphorylation (pS) at S1416[3]. The SH2-like domain binds to phosphotyrosines on multiple receptor tyrosine kinases upon activation[4]. The tyrosine phosphorylation sites (pY) are Y1764 and Y1798, two sites previously shown to bind and regulate PI3K[5]. Two serine phosphorylation sites (S1674 and S1689) flank the GBA motif and have been shown to modulate G protein binding and activation[6,7], although activation occurs *in*

vitro and in cells in the absence of phosphorylation[8-10]. *Bottom*, alignment of KB-752 peptide and GBA motif sequences. The background was shaded black if the residue was identical in more than 50% of the sequences and grey if it was similar in more than 50% of the sequences. The consensus sequence is shown below (ψ= hydrophobic, x=any). The invariable phenylalanine (F), critical for G protein binding, is colored red. GBAS-1 corresponds to GBA and SPK containing-1[11].

B. Overlay of Gαi1-GDP alone[12] (grey, PDB:1GDD) and Gαi-GDP in complex with the GIV-related GEF peptide KB-752[13] (blue, PDB:1Y3A). *Left,* KB-752 (orange ribbon) binds to a groove formed by the α3 helix and the Switch II of Gαi1, a region separated from the nucleotide (in sticks) binding pocket. Switch II is disordered in the structure of Gαi1-GDP alone whereas it forms an α-helix when KB-752 is bound. *Right*, the structure of the nucleotide binding pocket in the Gαi1/KB-752 structure is almost identical to the nucleotide binding pocket of Gαi1-GDP alone. Critical residues making contacts with the nucleotide are displayed in stick representation. Both backbone and side chains of amino acids forming the nucleotide binding pocket overlap closely in the two structures.

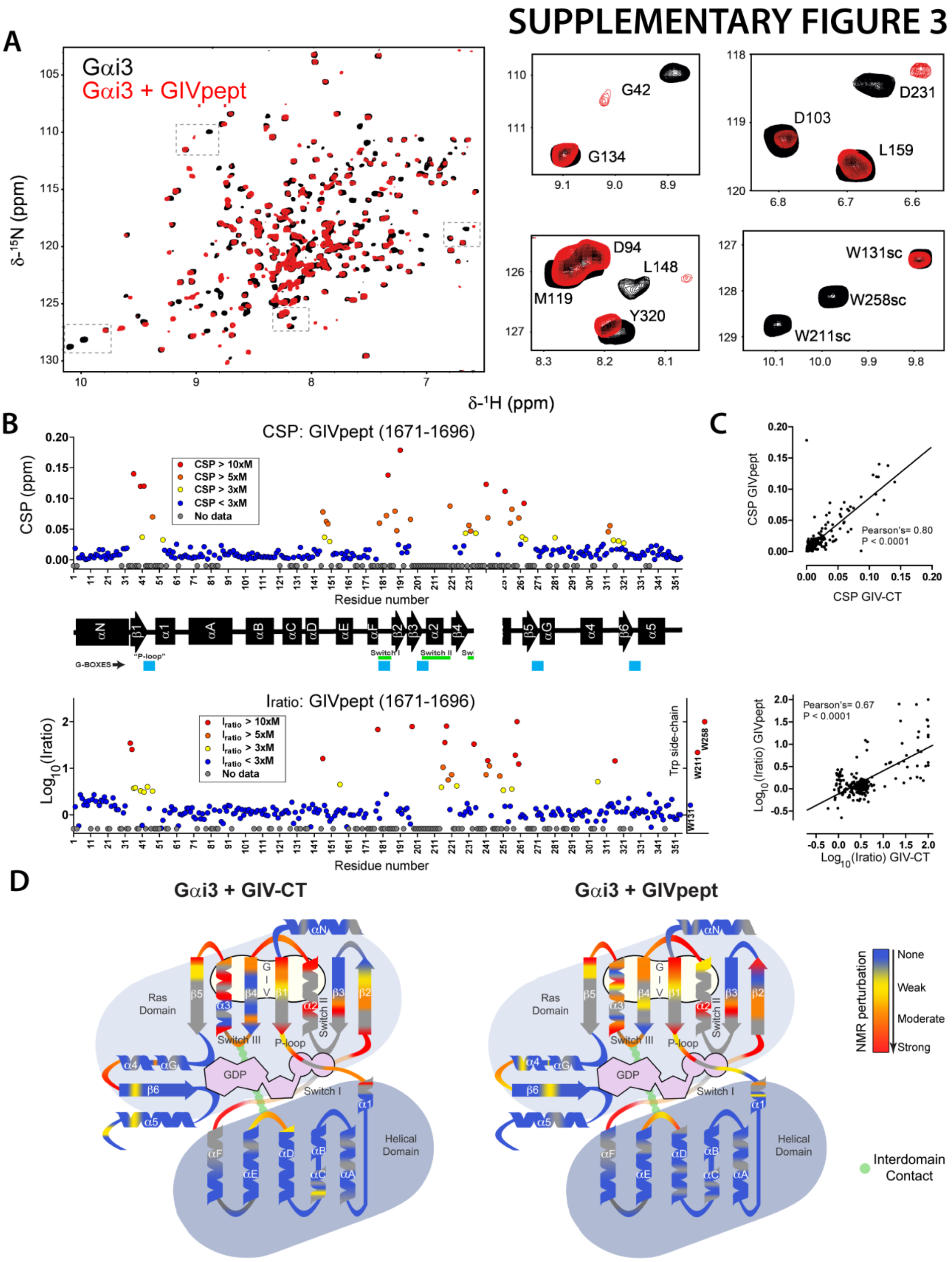

Supplementary Fig. 3. Gαi3 NMR signal perturbations upon binding of a 26-mer GIV-derived peptide (GIVpept) are similar to those observed upon GIV-CT binding.

A. Overlay of ^{1}H-^{15}N TROSY spectra of Gαi3 free and bound to GIVpept. ^{1}H-^{15}N TROSY spectra (backbone amide region) of ^{2}H,^{13}C,^{15}N-Gαi3 in the absence (black) or presence of a GIV-derived peptide (1671-1696, GIVpept) (red). *Right*, selected regions from the overlaid

spectra depicting the perturbations in the same Gαi3 signals induced by GIV binding as shown in Figure 2A.

B. Quantification of GIV-induced NMR perturbations on Gαi3. Chemical shift (CSP, top graph) or intensity perturbations (I_{ratio}, bottom graph) of the backbone amide signals of the TROSY NMR spectra in panel A. Red, orange and yellow circles indicate residues undergoing perturbations larger than 10, 5 or 3 times the median (M), respectively. Blue circles indicate Gαi3 residues with perturbations smaller than 3 times the median (M) and grey circles residues for which no reliable NMR measurement could be made. The horizontal black bar in the middle depicts the secondary structure elements of Gαi3 and is annotated with the position of the 3 switch regions (green) that undergo dramatic conformational changes upon GTP binding and the 5 conserved G-box sequences (blue) that mediate nucleotide binding.

C. Correlation scatter plots for CSP (top) and I_{ratio} (bottom) induced by GIV-CT and GIVpept. Both CSP and I_{ratio} for GIV-CT (1660-1870) and GIVpept (1671-1696) show good correlation as indicated by Pearson's coefficients >0.65.

D. Schematic representation of NMR perturbations on Gαi3 induced by GIV-CT (left) or GIVpept (right) binding. The diagram design and color coding is same as in Fig. 2C. The cartoon on the left (GIV-CT) is a duplication of Fig. 2C shown here for comparison.

SUPPLEMENTARY FIGURE 4

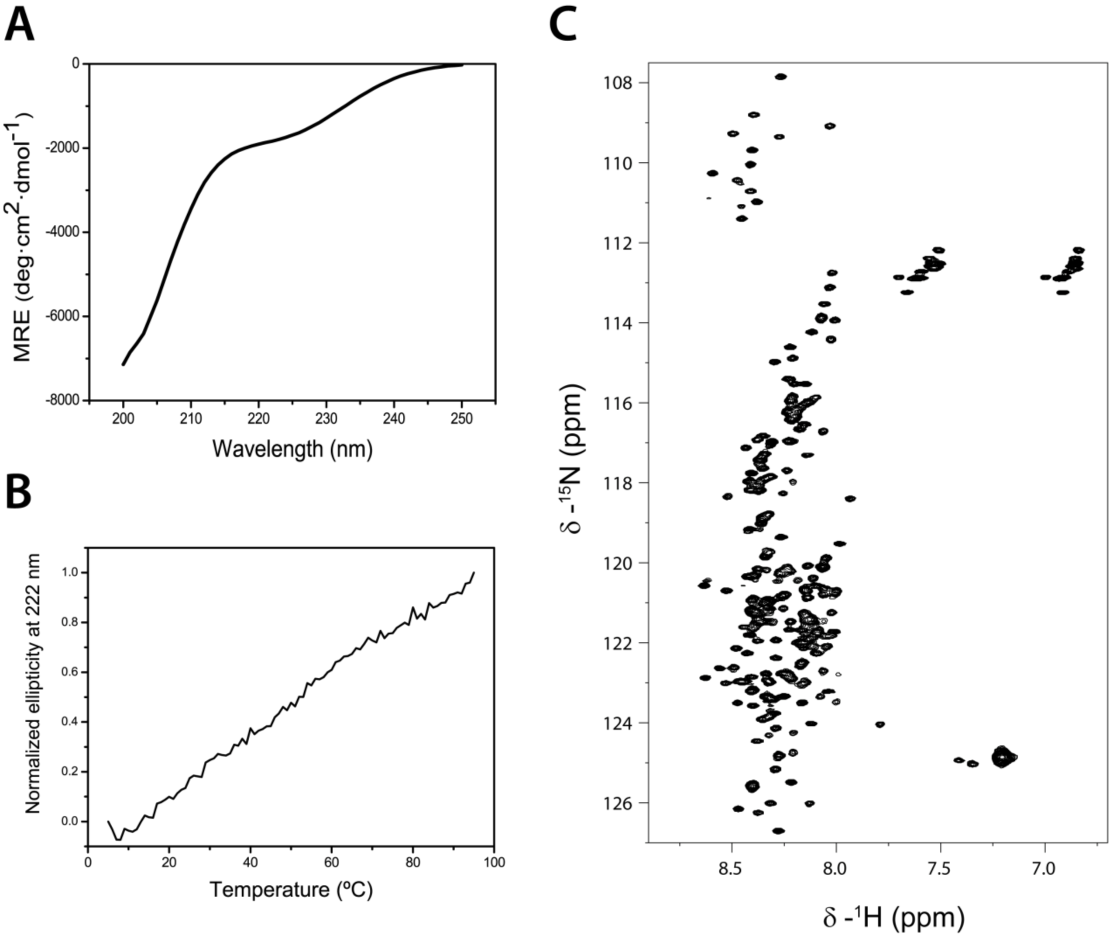

Supplementary Fig. 4. The C-terminal fragment of GIV is intrinsically disordered.

A. Circular dichroism of His-tagged human GIV residues 1660-1870. The spectrum was recorded at 25 ºC on a 8.4 μM sample in PBS pH 7.0 with 0.2 mM DTT.

B. Thermal denaturation of His-tagged human GIV residues 1660-1870. The ellipticity of the sample at 222 nm was measured between 5 and 95 ºC at 1 ºC intervals and normalized between 1 and 0.

C. NMR ^{1}H-^{15}N HSQC spectrum of His-tagged human GIV residues 1660-1870. The spectrum was recorded at 800 MHz and 25 ºC on a 125 μM sample in PBS pH 5.5 with 0.5 mM DTT. The signals in the region 7.0 - 7.5 ppm in the ^{1}H dimension and around 125 ppm in the ^{15}N dimension, corresponding to the arginine side chain NHε groups, are folded in the ^{15}N dimension.

SUPPLEMENTARY FIGURE 5

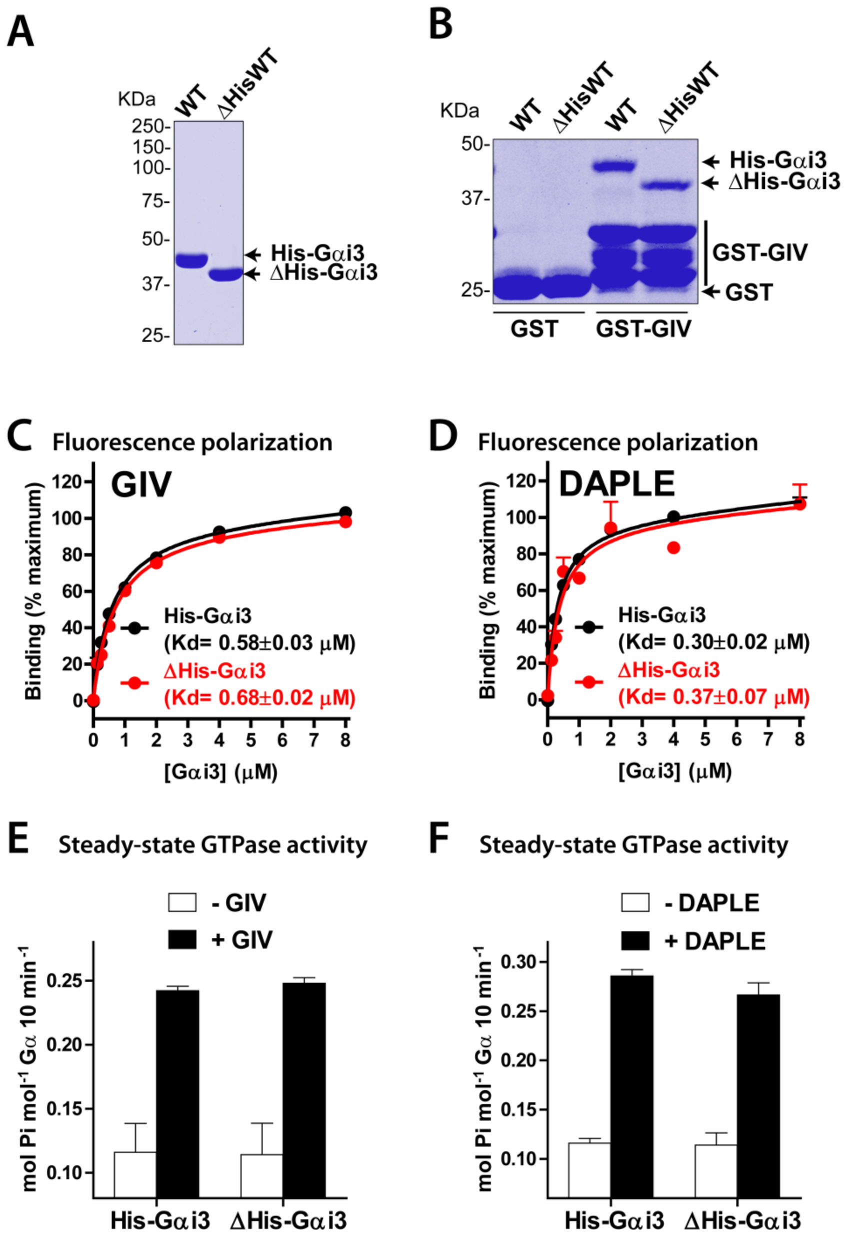

Supplementary Fig. 5. His-tagged human Gαi3 (His-Gαi3) has the same biochemical properties as Gαi3 after His-tag cleavage (ΔHis-Gαi3).

A. Purified His-Gαi3 and ΔHis-Gαi3. Five µg of each protein were run in an SDS-PAGE gel and stained with Coomassie blue.

B. A GIV C-terminal fragment binds equally to His-Gαi3 and ΔHis-Gαi3 in pulldown assays. Binding of His-Gαi3 and ΔHis-Gαi3 to GST or GST-GIV (residues 1671-1755) immobilized on glutathione-agarose beads was determined in pulldown assays. Resin-bound proteins were eluted, separated by SDS-PAGE and analyzed by Coomassie blue staining.

C. A GIV fragment corresponding to its GBA motif binds to His-Gαi3 and ΔHis-Gαi3 with the same affinity. Binding of fluorescein-labeled GIV (residues 1671-1701) peptide to His-Gαi3 (black) and ΔHis-Gαi3 (red) was determined by fluorescence polarization and fitted to a one site binding model to calculate the equilibrium dissociation constant (Kd) at room temperature. Mean ± S.E.M, n=3.

D. A DAPLE fragment corresponding to its GBA motif binds His-Gαi3 and ΔHis-Gαi3 with the same affinity. Binding of fluorescein-labeled DAPLE (residues 1662-1695) peptide to His-Gαi3 and ΔHis-Gαi3 was analyzed as in C. Mean ± S.E.M, n=3.

E, F. His-Gαi3 and ΔHis-Gαi3 have the same steady-state GTPase activity, which is enhanced to the same extent by either GIV (E) or DAPLE (F). Steady-state GTPase activity of His-Gαi3 and ΔHis-Gαi3 in the absence (white) or presence (black) of GIV (E) or DAPLE (F). Mean ± S.E.M, n=3.

SUPPLEMENTARY FIGURE 6

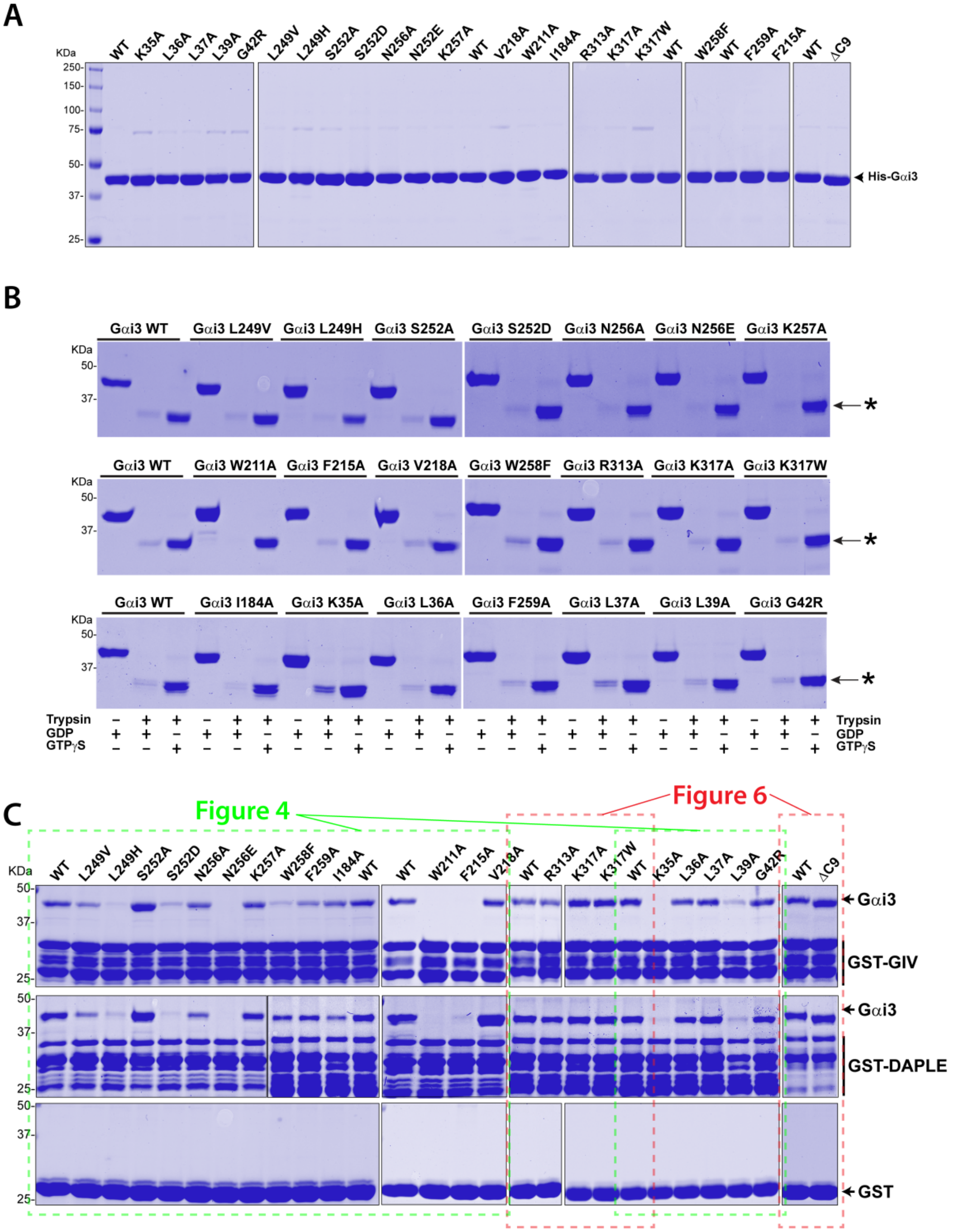

Supplementary Fig. 6. Quality controls of His-Gαi3 mutants used in this study and full dataset of pulldowns shown in Fig. 4 and Fig. 6.

A. Purified His-Gαi3 mutants. Five µg of each protein were run in an SDS-PAGE gel and stained with Coomasie blue.

B. All Gαi3 mutants used in this study adopt an active conformation upon GTPγS binding as determined by limited proteolysis assays. Each protein was incubated with GDP or GTPγS before limited digestion with trypsin. Proteins were run in an SDS-PAGE gel and stained with Coomassie blue. GDP-bound Gαi is readily digested by trypsin whereas active Gαi

generated by the binding of the non-hydrolyzable GTP analog GTPγS adopts a conformation that is resistant to trypsin digestion outside of a short N-terminal sequence which is cleaved off by trypsin[9]. All Gαi3 mutants adopt a trypsin-resistant conformation upon GTPγS binding (indicated by * on the right) to a similar extent as that observed for Gαi3 WT, indicating that the mutants do not have overt folding defects that preclude GTP binding and activation. Only Gαi3 L249H showed a modest decrease in trypsin protection but its steady-state GTPase activity was intact (see Fig. Supplementary 6B). One experiment representative of at least 3 is shown.

C. Full dataset with loading and negative controls for the pulldown results shown in Fig. 4 and Fig. 6. Binding of His-Gαi3 WT or mutants to GST-GIV (aa1671-1755) (top), GST-DAPLE (residues 1650-1880) (middle) or GST (bottom) immobilized on glutathione-agarose beads was determined in pulldown assays. Resin-bound proteins were eluted, separated by SDS-PAGE and analyzed by Coomassie blue staining. The multiple band patterns of GST-GIV and GST-DAPLE correspond to degradation products generated during protein purification. High sensitivity to proteolytic degradation is consistent with the disordered nature of these fragments (**Supplementary Fig. 4** and see reference[14]) but it is not a concern for the conclusions drawn about the binding of different Gαi3 mutants because (i) binding of Gαi3 WT to the same GST-GIV preparation (i.e., with identical proteolytic cleavage pattern) was used as an internal control for each gel/ experiment and, (ii) equivalent G protein binding is observed with preparations displaying less degradation products (**Fig. 1**). No binding to GST was detected. One experiment representative of at least 3 is shown. The green and red dashed boxes indicate the datasets corresponding to the results shown in Fig. 4 and Fig. 6, respectively.

SUPPLEMENTARY FIGURE 7

A

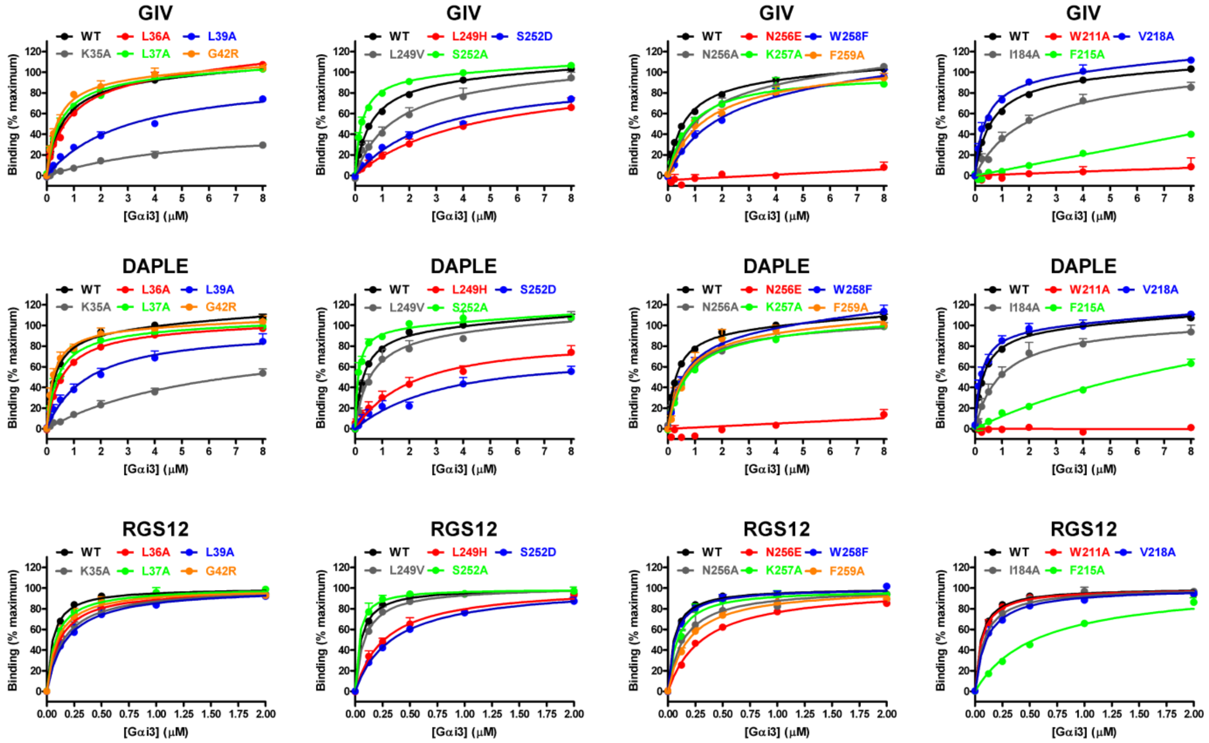

B

	BASAL GTPase activity		BASAL GTPγS binding	
	GTPase activity ± SD mol Pi mol⁻¹ Gα 10 min⁻¹	% of WT	GTPγS binding ± SD mol GTPγS mol⁻¹ Gα 10 min⁻¹	% of WT
WT	0.0118 ± 0.0016	100	0.0114 ± 0.0022	100
K35A	0.0128 ± 0.0026	109	ND	
L36A	0.0035 ± 0.0024	30	0.0038 ± 0.0003	33
L37A	0.0049 ± 0.0023	42	0.0072 ± 0.0002	63
L39A	0.0073 ± 0.0009	62	0.0070 ± 0.0005	61
G42R	0.0083 ± 0.0030	71	ND	
I184A	0.0058 ± 0.0014	50	0.0049 ± 0.0002	43
W211A	0.0089 ± 0.0011	76	0.0123 ± 0.0010	108
F215A	0.0214 ± 0.0057	182	ND	
V218A	0.0106 ± 0.0017	90	ND	
L249V	0.0138 ± 0.0037	117	ND	
L249H	0.0169 ± 0.0026	144	ND	
S252A	0.0129 ± 0.0019	110	ND	
S252D	0.0181 ± 0.0025	154	ND	
N256A	0.0120 ± 0.0021	102	ND	
N256E	0.0091 ± 0.0013	77	0.0095 ± 0.0006	83
K257A	0.0115 ± 0.0021	98	ND	
W258F	0.0150 ± 0.0017	127	ND	
F259A	0.0071 ± 0.0008	60	ND	
R313A	0.0115 ± 0.0011	98	ND	
K317A	0.0058 ± 0.0013	49	ND	
K317W	0.0029 ± 0.0003	25	ND	
ΔC9	0.0084 ± 0.0008	71	0.0083 ± 0.0010	72

C

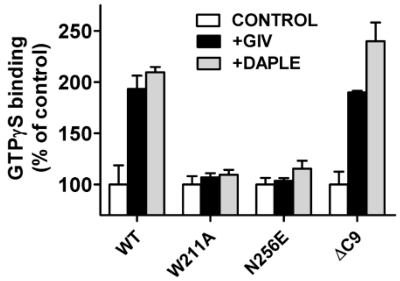

Supplementary Fig. 7. Full dataset of fluorescence polarization results shown in Fig. 4, basal GTPase activity of all Gαi3 mutants used in this study and GTPγS binding results for selected Gαi3 mutants.

A. Fluorescence polarization binding curves of GIV, DAPLE and RGS12 to different Gαi3 mutants used to calculate the Kd values shown in Fig. 4. Binding of fluorescein-labeled GIV (residues 1671-1701) (top), DAPLE (residues 1662-1695) (middle) or RGS12 (residues 1185-

1221) (bottom) peptides to His-Gαi3 WT or mutants was determined by fluorescence polarization and fitted to a one site binding model to determine the Kd as described in *Methods*. The result with His-Gαi3 WT is reproduced in every graph (black traces) to facilitate comparison with the mutants. Mean ± S.E.M, n=3-7.

B. GTPase and GTPγS binding activities for Gαi3 mutants used in this study. Basal GTPase and GTPγS binding activities of His-Gαi3 WT and the indicated mutants (first column) in the absence of GIV or DAPLE were determined as described in *Methods*. The second and fourth columns correspond to the raw GTPase and GTPγS binding activities, respectively (mean ± S.E.M, n≥3). The third and fifth columns correspond to GTPase and GTPγS binding activities expressed relative to WT (%). ND= Not determined. Mutants with GTPase activities below 65% of that of WT (marked in red in column two) were excluded from subsequent analyses of Gαi3 activation by GIV or DAPLE (they correspond to the *ND, not determined* mutants in the GTPase assays shown in Fig. 4 and Fig. 6). GTPase defects of selected mutants correlated well with defects in the basal GTPγS binding (column five), indicating impaired nucleotide binding rather impaired nucleotide hydrolysis.

C. Effect of GIV and DAPLE on the rate of GTPγS binding by selected Gαi3 mutants. GTPγS binding to His-Gαi3 WT or the indicated mutants in the absence (white) or presence of GIV (black) or DAPLE (grey) was determined as described in *Methods*. Mean ± S.E.M, n=3. The lack of GIV- or DAPLE-mediated activation for mutants W211A and N256E in GTPγS binding assays correlates with the lack of activation in steady-state GTPase assays shown in Fig. 4. Conversely, GIV and DAPLE enhance GTPγS binding to the Gαi3 ΔC9 mutant as efficiently as for WT, which correlates with the activation observed for the same mutant in steady-state GTPase assays shown in Fig. 6.

SUPPLEMENTARY FIGURE 8

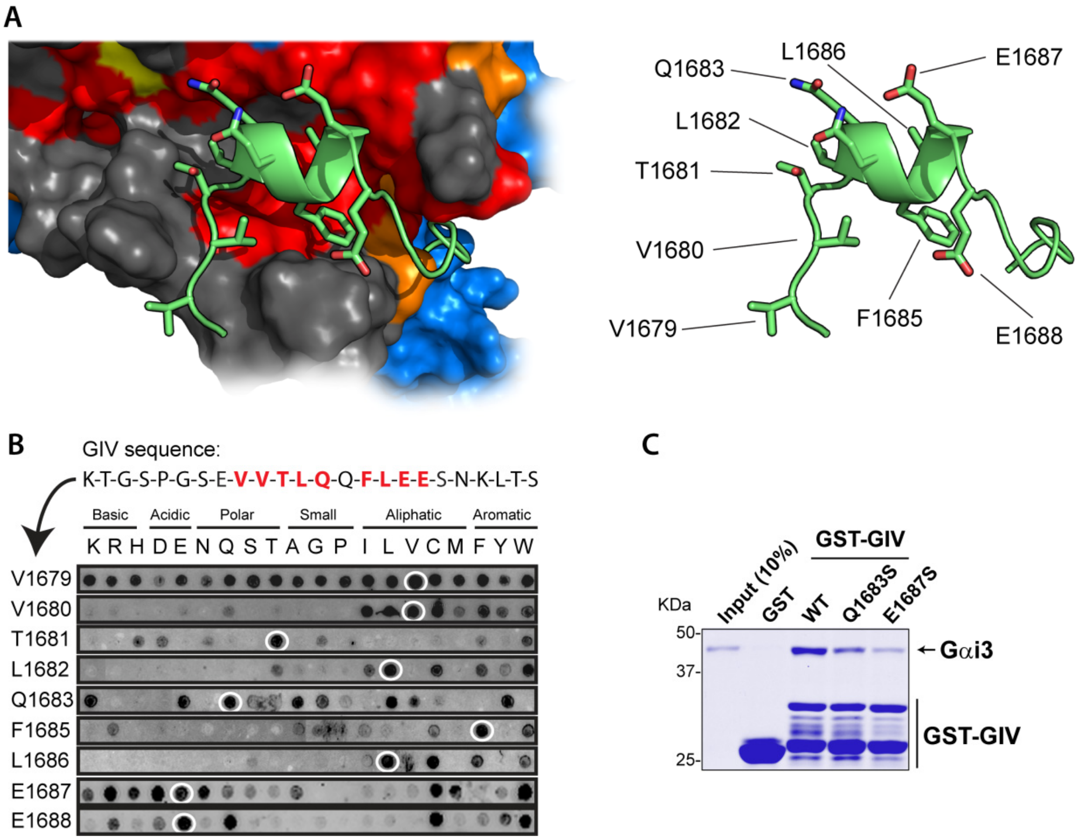

Supplementary Fig. 8. Characterization of the structural determinants of GIV required for Gαi3 binding. A. View of GIV docked on Gαi3 and localization of GIV residues selected for systematic peptide array mutagenesis. *Left*, Representation of the molecular model of Gαi3 bound to GIVpept (green ribbon). The surface of Gαi3 is colored based on NMR signal perturbations, and was generated as described in Fig. 3. *Right*, Gαi3 was removed from the image on the left and the residues (sticks) subjected to peptide array-based mutagenesis labeled.

B. Mutagenesis scanning of selected GIV residues in peptide arrays. 24-mer GIV peptides corresponding to the sequence shown on top were synthesized and immobilized on slides. Each one of the residues indicated in red in the sequence on top was substituted with every other natural amino acid. Each row corresponds to a series of mutants in which the residue indicated on the left is mutated to the amino acid type indicated on the top. Each spot is one peptide and the spots circled in white correspond to the wild-type peptide in each series. The immobilized peptides were probed in batch with purified Gαi3 and binding determined after sequential incubation with primary and secondary antibodies coupled to fluorescent probes. Similar results were obtained in two other experiments, and similar peptide content in all the spots was validated by Coomassie staining. See **Supplementary Note 2** for further discussion of these results in the context of the Gαi3 mutagenesis data.

C. Q1683S or E1677S mutation in GIV impairs Gαi3 binding in pulldown assays. Q1683S and E1677S were selected for validation in protein-protein binding experiments in solution (see **Supplementary Note 2** for the rationale of residue selection). Binding of His-Gαi3 WT or mutants to GST-GIV (residues 1671-1755) or GST was determined in pulldown assays. Resin-bound proteins were eluted, separated by SDS-PAGE and analyzed by Coomassie blue staining. The multiple band pattern of GST-GIV corresponds to degradation products generated during protein purification. No binding to GST was detected. One experiment of at least 3 is shown.

SUPPLEMENTARY FIGURE 9

Figure 1- Panel B

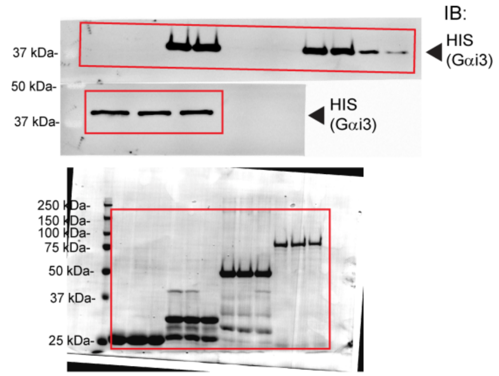

Supplementary Figure 1- Panel B

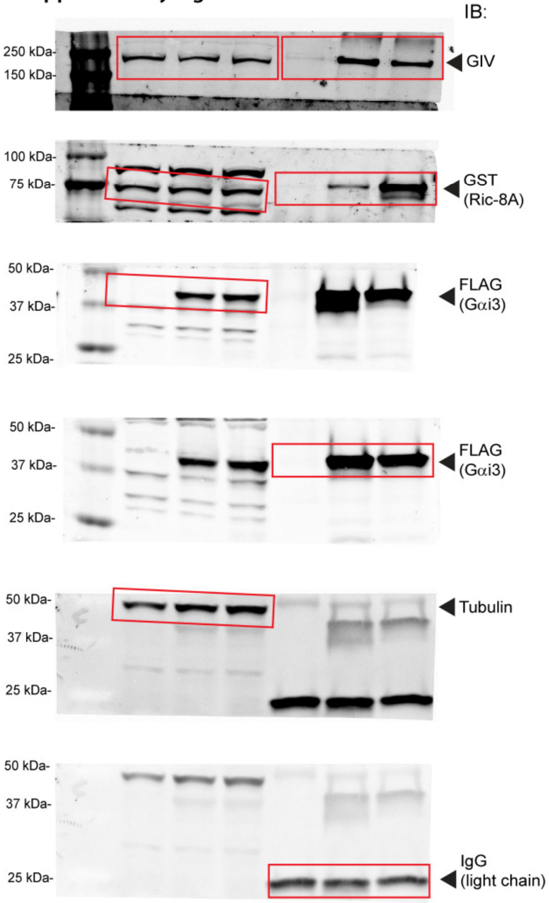

Figure 7- Panel B

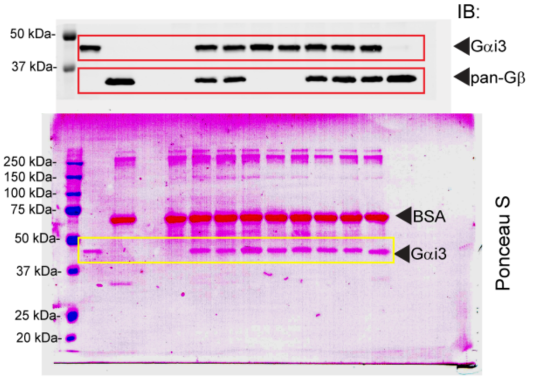

Figure 7- Panel E (left)

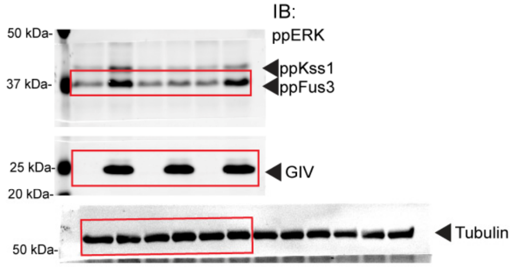

Figure 7- Panel E (right)

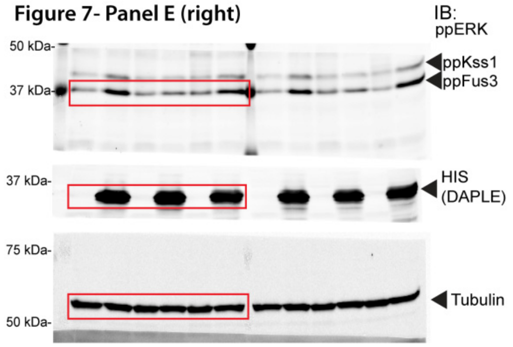

Figure 7- Panel F

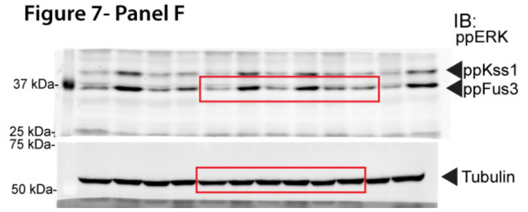

Supplementary Figure 5- Panel A

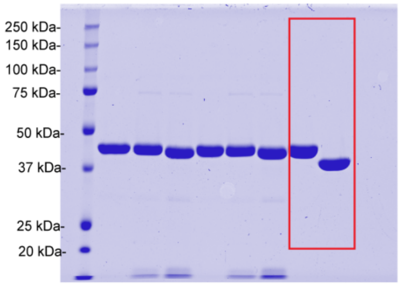

Supplementary Figure 5- Panel B

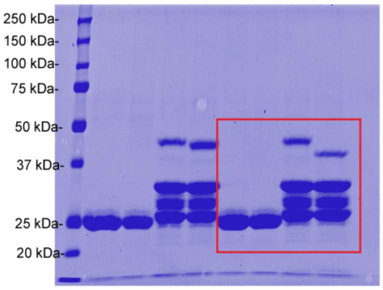

Supplementary Figure 6- Panel A (representative)

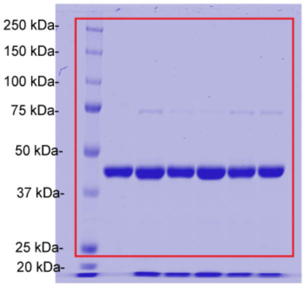

Supplementary Figure 6- Panel B (representative)

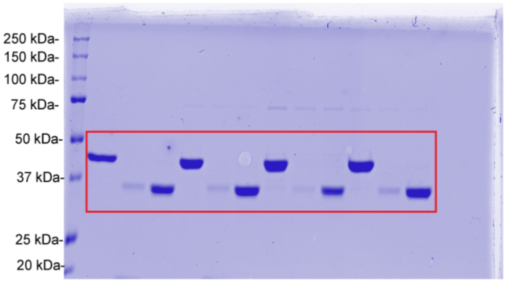

Supplementary Figure 6- Panel C top (representative)

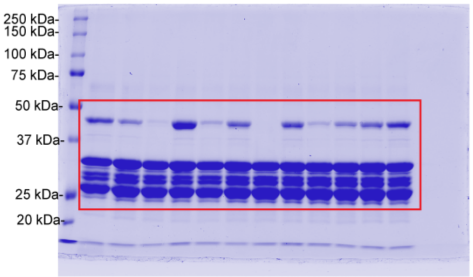

Supplementary Figure 6- Panel C middle (representative)

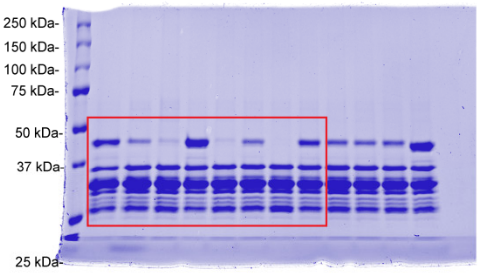

Supplementary Figure 6- Panel C bottom (representative)

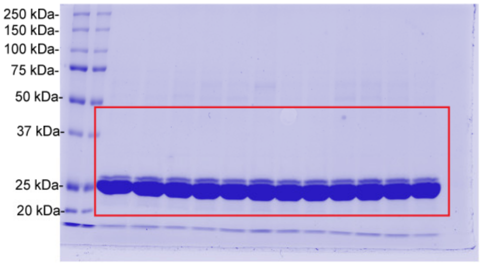

Supplementary Figure 8- Panel C

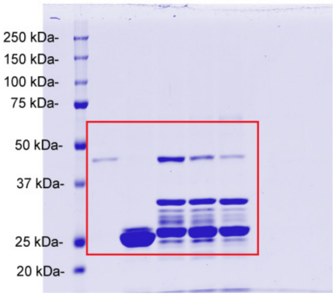

Supplementary Figure 9. Images of uncropped gels. Red boxes indicate the parts of that are shown in the corresponding final figures.

SUPPLEMENTARY NOTE 1

Rationale for the design of Gαi3 mutants- The mutants used in **Fig. 4** and **Fig.6** were designed based on the joint analysis of the NMR signal perturbations upon GIV binding (**Fig. 4, Supplementary Fig. 3**) and the *in silico* prediction of the contribution of individual amino acids to the energetics of the GIV-Gαi3 interaction (**Fig. 4A, 4B, Fig. 6A**). First, we looked for amino acids displaying NMR signal perturbations that were representative of different regions of Gαi3 (β1 strand, P-loop, α3 helix, α3/α5 loop, α4/β6 loop and SwII and SwI regions). Within this group, we selected amino acids that were predicted to stabilize the interaction with GIV (K35, W211, F215, L249, N256, W258, F259) or not (L36, L37, G42, I184, V218, S252, R313, K317). We also mutated L39 and K257, two residues for which no NMR information was available but were adjacent to other amino acids undergoing NMR signal perturbations. These were chosen to test the accuracy of our homology model and related *in silico* predictions. L39 was mutated because our model predicted it to stabilize GIV binding despite being adjacent to two amino acids (L37 and G40) not predicted to contribute to GIV binding (but displaying strong NMR signal perturbations). K257 was mutated because our model predicted it to not stabilize GIV binding despite being adjacent to two amino acids (N256 and W258) predicted to contribute to GIV binding and/or displaying strong NMR signal perturbations. The default mutation was to alanine (A) but in some cases residues were mutated to other amino acids based on a closer examination of the homology model because they were expected to have a more marked effect on binding. These include L249V, L249H, S252D, N256E and K317W. W258 was mutated to F to serve as an internal benchmark because the effect of this mutation on GIV binding has been extensively characterized by different techniques. W211A and F215A were also included as benchmarks because both of them have been shown to impair GIV[10], DAPLE[15] or Calnuc[16] binding using experimental approaches different from those described here.

SUPPLEMENTARY NOTE 2

Rationale and interpretation of the experiments shown in Supplementary Fig. 8- To further validate the results derived from the Gαi3 mutagenesis and support that our homology model faithfully represents the structural features of the GIV-Gαi3 interaction, we performed a systematic mutagenesis analysis of the GBA motif of GIV. We reasoned that those amino acid positions more directly involved in binding Gαi3 would be more sensitive to substitution by other amino acids and/or by the chemical nature of the side chain substitution. Nine positions in the region of the GBA motif were chosen for this analysis. These included positions predicted to

participate in (e.g., Q1683) or not (e.g., V1679), as well as positions already known to be important for binding as benchmarks (e.g., F1685[10,17]). Each position was mutated to every other natural amino acid to create a grid of 171 immobilized peptide variants that were probed in batch for Gαi3 binding. The results obtained through this approach complemented the results of the Gαi3 mutagenesis studies (**Fig. 4**) and validated the accuracy of our model. For example, F1685 has been repeatedly shown to be crucial for Gαi3 binding[10,17] and the peptide array revealed that its substitution by any other amino acid dramatically impairs G protein binding. Similarly, L1682 and L1686, two residues that together with F1685 dock onto the hydrophobic cleft lined by Gαi3 residues W211 and F215 based on our model, are also very sensitive to mutation. The GIV-Gαi3 interaction only tolerates some substitutions for other hydrophobic residues in these positions, which is also consistent with our model. Mutation of E1688 to A has been previously shown to disrupt GIV binding to Gαi3[10] and the peptide array indicates that the GIV-Gαi3 interaction tolerates E1688 substitution only by some amino acids in this position. Replacement of T1681 by essentially any other amino acid also disrupts Gαi3 binding. This is likely explained by the role of this residue in maintaining the α-helical structure of the GBA motif, as previously discussed by Johnston et al. based on the structure of GBA-related peptide KB-752 in complex with Gαi1[13]. The results with the two adjacent positions V1679 and V1680 provide additional insights supporting the validity of our model. Consistent with our model showing that V1679 is predominantly solvent exposed, the peptide array revealed that this position can be replaced by virtually any other amino acid without impairing Gαi3 binding. In contrast, V1680 can only be replaced by other hydrophobic residues without affecting Gαi3 binding, which is consistent with our model showing that this residue is buried in a hydrophobic environment of Gαi3. Many mutations in positions Q1683 and E1687 also impair Gαi3 binding, which is consistent with our results with Gαi3 mutants (**Fig. 4**). For example, Q1683 is located in the vicinity of Gαi3 L249 and E1687 in the vicinity of Gαi3 S252/N256. Because the role of these two positions of GIV on Gαi3 binding has not been previously investigated, we performed additional validation experiments to evaluate protein-protein binding in solution. For this, we mutated each one of these residues to serine (Q1683S or E1687S). We chose serine because it is one of the substitutions that impair Gαi3 binding in the peptide array format yet it is moderately conservative. We found that both mutants impaired GIV binding to Gαi3 as determined by GST pulldown assays (**Supplementary Fig. 8C**).

SUPPLEMENTARY REFERENCES

1. Wu, Y.L., Hooks, S.B., Harden, T.K. & Dohlman, H.G. Dominant-negative inhibition of pheromone receptor signaling by a single point mutation in the G protein alpha subunit. *J Biol Chem* **279**, 35287-97 (2004).
2. Simpson, F., Martin, S., Evans, T.M., Kerr, M., James, D.E., Parton, R.G., Teasdale, R.D. & Wicking, C. A novel hook-related protein family and the characterization of hook-related protein 1. *Traffic* **6**, 442-58 (2005).
3. Enomoto, A., Murakami, H., Asai, N., Morone, N., Watanabe, T., Kawai, K., Murakumo, Y., Usukura, J., Kaibuchi, K. & Takahashi, M. Akt/PKB regulates actin organization and cell motility via Girdin/APE. *Dev Cell* **9**, 389-402 (2005).
4. Lin, C., Ear, J., Midde, K., Lopez-Sanchez, I., Aznar, N., Garcia-Marcos, M., Kufareva, I., Abagyan, R. & Ghosh, P. Structural basis for activation of trimeric Gi proteins by multiple growth factor receptors via GIV/Girdin. *Mol Biol Cell* **25**, 3654-71 (2014).
5. Lin, C., Ear, J., Pavlova, Y., Mittal, Y., Kufareva, I., Ghassemian, M., Abagyan, R., Garcia-Marcos, M. & Ghosh, P. Tyrosine phosphorylation of the Galpha-interacting protein GIV promotes activation of phosphoinositide 3-kinase during cell migration. *Sci Signal* **4**, ra64 (2011).
6. Bhandari, D., Lopez-Sanchez, I., To, A., Lo, I.C., Aznar, N., Leyme, A., Gupta, V., Niesman, I., Maddox, A.L., Garcia-Marcos, M., Farquhar, M.G. & Ghosh, P. Cyclin-dependent kinase 5 activates guanine nucleotide exchange factor GIV/Girdin to orchestrate migration-proliferation dichotomy. *Proc Natl Acad Sci U S A* **112**, E4874-83 (2015).
7. Lopez-Sanchez, I., Garcia-Marcos, M., Mittal, Y., Aznar, N., Farquhar, M.G. & Ghosh, P. Protein kinase C-theta (PKCθ) phosphorylates and inhibits the guanine exchange factor, GIV/Girdin. *Proc Natl Acad Sci USA* **110**, 5510-5515 (2013).
8. Parag-Sharma, K., Leyme, A., DiGiacomo, V., Marivin, A., Broselid, S., Ramachandran, S., Cerione, R.A. & Garcia-Marcos, M. Membrane recruitment of the non-receptor protein GIV/Girdin is sufficient for activating heterotrimeric G proteins *J Biol Chem* **In press**(2016).
9. Garcia-Marcos, M., Ghosh, P., Ear, J. & Farquhar, M.G. A structural determinant that renders G alpha(i) sensitive to activation by GIV/girdin is required to promote cell migration. *J Biol Chem* **285**, 12765-77 (2010).
10. Garcia-Marcos, M., Ghosh, P. & Farquhar, M.G. GIV is a nonreceptor GEF for G alpha i with a unique motif that regulates Akt signaling. *Proc Natl Acad Sci U S A* **106**, 3178-83 (2009).
11. Coleman, B.D., Marivin, A., Parag-Sharma, K., DiGiacomo, V., Kim, S., Pepper, J.S., Casler, J., Nguyen, L.T., Koelle, M.R. & Garcia-Marcos, M. Evolutionary Conservation of a GPCR-Independent Mechanism of Trimeric G Protein Activation. *Mol Biol Evol* **33**, 820-37 (2016).
12. Mixon, M.B., Lee, E., Coleman, D.E., Berghuis, A.M., Gilman, A.G. & Sprang, S.R. Tertiary and quaternary structural changes in Gi alpha 1 induced by GTP hydrolysis. *Science* **270**, 954-60 (1995).
13. Johnston, C.A., Willard, F.S., Jezyk, M.R., Fredericks, Z., Bodor, E.T., Jones, M.B., Blaesius, R., Watts, V.J., Harden, T.K., Sondek, J., Ramer, J.K. & Siderovski, D.P. Structure of Galpha(i1) bound to a GDP-selective peptide provides insight into guanine nucleotide exchange. *Structure* **13**, 1069-80 (2005).

14. Oates, M.E., Romero, P., Ishida, T., Ghalwash, M., Mizianty, M.J., Xue, B., Dosztanyi, Z., Uversky, V.N., Obradovic, Z., Kurgan, L., Dunker, A.K. & Gough, J. D(2)P(2): database of disordered protein predictions. *Nucleic Acids Res* **41**, D508-16 (2013).
15. Aznar, N., Midde, K.K., Dunkel, Y., Lopez-Sanchez, I., Pavlova, Y., Marivin, A., Barbazan, J., Murray, F., Nitsche, U., Janssen, K.P., Willert, K., Goel, A., Abal, M., Garcia-Marcos, M. & Ghosh, P. Daple is a novel non-receptor GEF required for trimeric G protein activation in Wnt signaling. *Elife* **4**(2015).
16. Garcia-Marcos, M., Kietrsunthorn, P.S., Wang, H., Ghosh, P. & Farquhar, M.G. G Protein binding sites on Calnuc (nucleobindin 1) and NUCB2 (nucleobindin 2) define a new class of G(alpha)i-regulatory motifs. *J Biol Chem* **286**, 28138-49 (2011).
17. Garcia-Marcos, M., Ghosh, P. & Farquhar, M.G. GIV/Girdin transmits signals from multiple receptors by triggering trimeric G protein activation. *J Biol Chem* **290**, 6697-704 (2015).
